# Prepare for the Future: Dissecting the Spike to Seek Broadly Neutralizing Antibodies and Universal Vaccine for Pandemic Coronaviruses

**DOI:** 10.3389/fmolb.2020.00226

**Published:** 2020-09-01

**Authors:** Luca Vangelista, Massimiliano Secchi

**Affiliations:** ^1^Department of Biomedical Sciences, Nazarbayev University School of Medicine, Nur-Sultan, Kazakhstan; ^2^Diabetes Research Institute, IRCCS San Raffaele Hospital, Milan, Italy

**Keywords:** COVID-19, SARS-CoV-2, coronavirus, spike, conserved regions, broadly neutralizing antibodies, universal vaccine, antibody-dependent enhancement

## Abstract

Learning from the lengthy fight against HIV-1, influenza, and Ebola virus infection, broadly neutralizing antibodies (bnAbs), directed at conserved regions of surface proteins crucial to virus entry (Env, hemagglutinin, and GP, respectively), are an essential resource for passive as well as active immunization. Rare in their emergence and antigen recognition mode, bnAbs are active toward a large set of different viral strains. Isolation, characterization and production of bnAbs lead to their possible use in passive immunotherapy and form the basis for an educated effort in the development of vaccines for universal coverage. SARS-CoV-2-specific antibodies targeting the spike receptor binding domain (RBD) may lead to antibody dependent enhancement (ADE) of infection, possibly hampering the field of vaccine development. This perspective points to the identification of conserved regions in the spike of SARS-CoV-2, SARS-CoV, and MERS-CoV through investigation, dissection and recombinant production of isolated moieties. These spike moieties should be capable of independent folding and allow the detection as well as the elicitation of bnAbs, thus setting the basis for an effective passive immunotherapy and the development of a universal vaccine against human epidemic coronaviruses (HCoVs). SARS, MERS and, most of all, COVID-19 demonstrate that humanity is the target of HCoV, preparedness for future hits is thus no longer an option.

## Introduction

A worldwide effort to combat COVID-19, the pandemic caused by SARS-CoV-2, includes the repurposing of different drugs to attempt pharmacological treatment of the infection and a large number of vaccine trials (Fauci et al., 2020). Protective antibodies present in the blood of convalescent individuals that won the fight against SARS-CoV-2 also attract a lot of attention (Chen et al., 2020; Tanne, 2020). During viral infections, seldom individuals produce broadly neutralizing antibodies (bnAbs), rare molecular entities that have the capacity to provide protection against a large number of viral strains. In HIV-1, influenza, and Ebola virus infection, several bnAbs proved to be extremely potent and effective at inhibiting virus entry (Kallewaard et al., 2016; King et al., 2019; Stephenson et al., 2020). This perspective aims to illustrate how searching for bnAbs in COVID-19 may turn out to be more relevant than just an option.

In the case of human epidemic coronaviruses (HCoVs) infection, antibody-dependent enhancement (ADE) has been observed for SARS-CoV and MERS-CoV. ADE could explain some peculiarities of COVID-19 immunopathology and may seriously hamper vaccination attempts (Huisman et al., 2009; Cao, 2020; Tetro, 2020). Caution is thus required when aiming at provoking an antibody immune response toward coronaviruses, as this response could either block the infection or enhance it (Iwasaki and Yang, 2020). A dramatic example of ADE induced by cross-reactive antibodies occurs in flaviviruses in which one serotype can predisposes to more severe infection by another serotype, and sequential infection of Zika and dengue viruses can generate ADE (Rey et al., 2018). ADE was not predicted in a vaccination campaign against dengue that resulted in generalized vaccine hesitancy (Fatima and Syed, 2018).

Coronavirus spike is a surface protein, key to target cells for entry through specific receptor recognition. Spike protein from both SARS-CoV and SARS-CoV-2 determine viral tropism by recognizing angiotensin converting enzyme 2 (ACE2). Differences in amino acid sequence at SARS-CoV-2 spike receptor binding domain (RBD), as compared to SARS-CoV, significantly increased its affinity for human ACE2 (Lan et al., 2020; Wrapp et al., 2020; Wang Q. et al., 2020; Yan et al., 2020). While SARS-CoV-2 RBD has been employed as an obvious and immediate target for vaccine development worldwide, the high variability within the receptor binding motif (RBM) would (in the case of a successful vaccine) most likely allow specific protection for SARS-CoV-2 but not automatically grant broad protection toward other HCoVs possibly emerging in the future. Thus, using the spike RBD for vaccination may lead to unwanted ADE effects and is limited to strain specificity.

Humanity urgently needs to counteract COVID-19 and the successful development of a vaccine would mark a victory over this pandemic. However, safety needs to remain a crucial criterion and the possibility to develop a vaccine protecting against several epidemic HCoVs would set a different standard of preparedness, averting future catastrophes of COVID-19 magnitude.

## Learning From Other Pandemic Viruses

**FIGURE 1:**
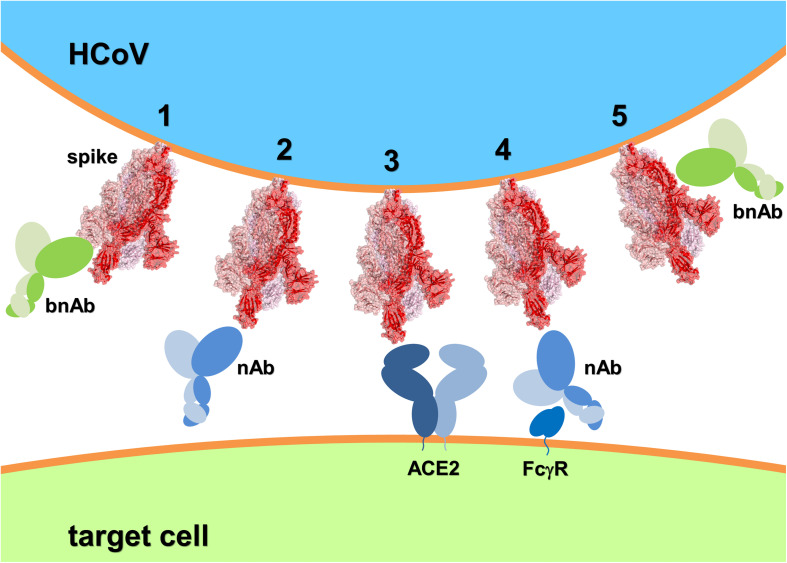
Schematic of HCoV spike recognition by bnAbs capable of freezing the spike conformational dynamics and inhibit receptor binding and membrane fusion (**1** and **5**, respectively); nAbs competing with the HCoV receptor **(2)** but presenting the risk of ADE through molecular mimicry and FcγR binding **(4)**; and the HCoV receptor **(3)**. The SARS-CoV-2:ACE2 system has been depicted as HCoV example. Spike 3D structure was generated using PyMOL from PDB entry 6VYB (Walls et al., 2020) and represented in ribbon and transparent surface (the three monomers have different shades of red).

Focusing on the progress of bnAbs recognizing the spike entry molecular machinery of different pandemic-prone viruses (such as HIV-1, influenza, and Ebola) may prove instrumental for the search of HCoV spike-specific bnAbs (Figure 1).

HIV-1 infection is a dramatic example of a long-lasting pandemic for which a vaccine is still awaited. Once the infection is established, HIV-1 can evade the host immune response by genetic variation and a wealth of different molecular tricks. Antibodies induced by both natural HIV-1 infection and vaccine candidates are generally not of sufficient quality to establish protection. A small number of HIV-1-infected individuals develop bnAbs that protect from HIV-1 strains of diverse genetic background with remarkable potency. These inspire immunization with sophisticated vaccines aimed at presenting Env constant regions with the goal of eliciting bnAbs (Stephenson et al., 2020). Env is a gp120-gp41 trimer protected by a glycan shield. Binding of gp120 to cellular CD4 and CCR5 (or CXCR4) induces conformational changes ultimately leading to gp41-mediated membrane fusion. Despite its remarkable variability, Env contains several conserved regions that can be targeted by bnAbs (Stephenson et al., 2020). A first generation of bnAbs presented relatively low potency, HIV-1 escape variants and self-reactivity. Nevertheless, b12 unveiled the vulnerable CD4 binding site (CD4bs), 2G12 the V3-glycan site and 4E10 and 2F5 the membrane-proximal external region (MPER). Technological advances allowed the isolation of a second generation of bnAbs from infected individuals that revealed new vulnerable Env epitopes: the V1/V2-glycan site (PG9, PGT145), the gp120/gp41 interface (35O22, 8ANC195), the fusion domain (VRC34.01, ACS202) and the silent face (VRC-PG05, SF12); and more bnAbs recognized the CD4bs (VRC01, N6, N49P7), the V3-glycan site (PGT121, 10-1074) and the MPER (10E8) (Wang and Zhang, 2020). The Env epitopes and conformational states recognized by these bnAbs provided unprecedented opportunities for HIV-1 vaccine design. The first strategy consisted in a soluble trimeric Env (BG505 SOSIP.664) that became the prototypic native-like Env as it displayed multiple epitopes recognized by bnAbs, however, lack of V3-glycan site recognition by bnAbs led to the use of different variants (Sanders and Moore, 2017). In addition to protein mimicry, glycan mimicry is necessary to elicit anti-glycan bnAbs, thus knowledge of the detailed composition of the Env glycan shield is essential for HIV-1 vaccine design. Overall, the elicitation of bnAbs is a major challenge in HIV-1 vaccine development as HIV-1 bnAbs generally have a long CDRH3 (Seabright et al., 2019).

Influenza virus trimeric hemagglutinin (HA) spikes mediate attachment to host cell sialic acid. Currently, seasonal influenza A and B vaccination needs annual re-formulation and re-administration due to virus antigenic drift, however, this approach does not shield against the possible emergence of pandemic strains due to antigenic shift or adaptive mutation. This threat has been largely confirmed by the 1918, 1957, 1968, and 2009 pandemics and points to the need for a universal influenza vaccine. Worldwide efforts gradually unfolded into the identification of constant regions of HA (the stalk and hidden epitopes on the head) as crucial hotspots and the discovery of bnAbs recognizing those regions. These antibodies are the key for universal protection against influenza. Blood samples analysis collected from influenza virus-infected or immunized individuals confirmed that most bnAbs target HA (Vogel and Manicassamy, 2020). In its trimeric conformation, each HA monomer is composed of the globular head domain formed by HA1 subunits and a long stem or stalk domain formed by HA2 subunits responsible of the fusion between viral and endosomal membrane. The HA1 region is highly immunogenic and variable and antibodies directed against this domain are usually strain-specific with strong virus-neutralizing activity exerted by preventing receptor binding (Krammer, 2019). Only few anti-HA head domain bnAbs have been described (Wang Y. et al., 2020). Isolated from a healthy donor, Flu-A20 revealed a new conserved hidden epitope (in the HA head at the trimer interface) and neutralized nearly all influenza A subtypes by disrupting the integrity of the HA trimer (Bangaru et al., 2019). Recently, H7 was found to interact with another HA head hidden epitope that is only partly and transiently exposed in the pre-fusion conformation. These findings suggest that the HA trimer possesses a conformational dynamics in which different pre-fusion conformations (breathing) briefly expose cryptic epitopes that can be targeted by protective antibodies (Turner et al., 2019). This breathing phenomenon has also been observed in other viruses, including HCoV spike, and could help to identify new conserved sites of vulnerability (Yuan et al., 2017; Wrapp et al., 2020). Conversely, antibody binding to the stalk domain prevents membrane fusion and leads to the trapping of the virus in the endosome. Stalk-directed antibodies may also bind to newly expressed HA proteins on the cell surface and interfere with viral budding. In contrast to the HA1 head, the HA2 stalk domain is highly conserved among different influenza virus subtypes and is considered as an important target for universal vaccine development (Jang and Seong, 2019). The stalk-specific bnAbs MHAA4549A and MEDI8852 target almost all HA types and groups, while CR9114 recognizes a conserved epitope in the HA stem and protects against lethal challenges with the influenza A and B viruses (Kallewaard et al., 2016; Sedeyn and Saelens, 2019). However, the stalk is less immunogenic than the head and bnAbs directed to the stalk are generally less potent than the head-specific antibodies. In addition to direct neutralization, HA stem-specific bnAbs usually require FcγR-mediated effector cell function (Vanderven and Kent, 2020). The development of a vaccine that promotes the generation of anti-stem antibodies is inherently difficult, yet two such vaccines are currently in development: one based on chimeric HA and one on headless HA (Krammer, 2016). In the chimeric HA vaccine, combinations of the same stalk with different head domains are administered sequentially, possibly disrupting the immunodominance of the head and directing the immune response to the stalk.

In the trimeric GP of Ebola virus each monomer is composed of GP1 and GP2 subunits. GP1 mediates host cell attachment and receptor recognition, whereas GP2 mediates membrane fusion. After virus internalization into target cells, host cathepsins remove the glycan cap and mucin-like domain of GP1 to generate the cleaved GP intermediate (GP_CL_) and expose the RBD for its interaction with the endosomal Niemann-Pick C1 receptor, inducing GP2 conformational rearrangements and membrane fusion (King et al., 2019). Trimeric GP of Zaire ebolavirus (EBOV) is the key component of the vaccine Ervebo and the unique target of nAbs (Saphire, 2020). EBOV caused deadly outbreaks in West Africa (2013–2016) and in the Democratic Republic of Congo (2018-ongoing). During the latter, treatments with mAb114 and mAb cocktails Zmapp and REGN-EB3 showed a reduction in mortality rates, proving that nAbs can be effective against Ebola virus (Mulangu et al., 2019). However, there is no vaccine or therapeutics available for human epidemic Bundibugyo (BDBV) and Sudan (SUDV) ebolaviruses (Wolfe et al., 2020). Since EBOV discovery in 1976, ebolaviruses reemerged over 20 times with fatality rates varying from 25 to 90% (Malvy et al., 2019). The urgency to attain broad protection against Ebola virus led to the discovery and characterization of different bnAbs. The glycan cap and mucin like domains of GP1 show high sequence diversity, while GP2 is highly conserved among ebolaviruses (Saphire et al., 2018). ADI-15878, ADI-15742, and ADI-15946, isolated from a human survivor of the recent West Africa outbreak, interact with a conserved epitope of GP_CL_ in the endosomes preventing membrane fusion (Wec et al., 2017). The structure of ADI-15946 in complex with EBOV GP_CL_ showed that the antibody binds to a hydrophobic conserved pocket of GP that is usually occupied by the glycan cap. ADI-15946 neutralizes EBOV and BDVD, but is not protective against SUDV (West et al., 2019). EBOV-520 targets a critical epitope in the GP1-GP2 junction (Gilchuk et al., 2018), while CA45 binds the internal fusion loop of GP2 (Zhao et al., 2017). Data from HIV-1 gp41 aided the identification of a conserved site within ebolavirus GP2 that can be recognized by bnAbs, the HR2/MPER (Flyak et al., 2018). Recently 2G1, a GP2-specific bnAb isolated from a vaccinated individual, exhibited cross-neutralization against EBOV, BDVD and SUDV (Fan et al., 2020), opening the possibility to develop pan-ebolavirus vaccines and antibody cocktails (Gilchuk et al., 2020).

## Leading Edge for Epidemic HCoVs

Coronaviruses are enveloped viruses with a positive-stranded RNA. Severe syndrome-causing HCoVs infections of humans begun in 2002 with the SARS outbreak: initially seen as an exotic event in the co-evolution of humans and coronaviruses, with MERS-CoV as a second major hit, and the present COVID-19 pandemic, sufficient evidence mounted to predict that there will be more such pandemic-risk strains in the future. Epidemic HCoVs and endemic (mild syndrome-causing) HCoV-OC43 and HCoV-HKU1 are betacoronaviruses, while endemic HCoV-229E and HCoV-NL63 are alphacoronaviruses (Weiss, 2020). HCoV spike is divided into the S1 and S2 regions; S1 contains the RBD, the N-terminal domain (NTD) and other subdomains, while S2 contains the fusion machinery (including the fusion peptide and the HR1 and HR2 regions).

Human ACE2 is the receptor for both SARS-CoV and SARS-CoV-2, while the receptor recognized by MERS-CoV is human dipeptidyl peptidase 4 (DPP4) (Raj et al., 2013). Controversial evidence exists on the mode of ADE in HCoV, some studies suggest the use of RBD for vaccine development, while its exclusion is suggested by other investigations (Hotez et al., 2020). Necessarily, the search for broad neutralization of epidemic HCoVs needs to be directed to conserved regions distal to the spike RBM of the RBD. A specific vaccine for SARS-CoV-2 is not yet a certainty and a drug will not necessarily be successfully repurposed to confront COVID-19 (Chary et al., 2020; Lurie et al., 2020; Sanders et al., 2020). To date, vaccine candidates for SARS-CoV and MERS-CoV did not reach final approval. Despite the general optimism, it is yet unknown whether inherent drawbacks might prevent a SARS-CoV-2 vaccine to be produced (Thorp, 2020b). ADE occurs when non-neutralizing antibodies are produced by the host immune system, or when neutralizing antibodies are present at concentrations suboptimal for neutralization. ADE is mediated by FcγR-bearing cell recognition of antibody-virus complexes, consequently, increase of pro-inflammatory cytokines and activation of immune cells lead to immunopathology (Iwasaki and Yang, 2020). This classic ADE occurs also in HCoVs infection, however, an additional mechanism has recently been described (Walls et al., 2019; Wan et al., 2020). Two mAbs (Mersmab1 and S230) respectively recognize MERS-CoV and SARS-CoV spike RBD exclusively in its standing-up orientation, a fusion-prone conformation. Thus, Mersmab1 and S230 act in a functional mimicry mode that triggers spike’s conformational change and membrane fusion. As a result, HCoV can infect immune cells through ADE triggered by receptor mimicking antibodies. A third mAb, LCA60, recognizes MERS-CoV RBD in its lying-down position an orientation that does not trigger the conformational change leading to membrane fusion (Walls et al., 2019; Wan et al., 2020). Antibodies capable to neutralize HCoV through the blockade of conformational changes and membrane fusion catalysis are less likely to induce ADE and could possibly recognize the spike on regions key to broad specificity (Figure 1). Promising evidence in this direction comes from 5F9 (NTD), G2 (NTD), and G4 (connector domain between HR1 and HR2) against MERS-CoV (Xu et al., 2019); 1F8 (HR1) and 5E9 (HR2) against SARS-CoV (Elshabrawy et al., 2012); and 47D11 that recognizes a conserved region of the RBD distal to the RBM in SARS-CoV-2 spike (Wang C. et al., 2020). Similarly, CR3022 and S309 (isolated from SARS-convalescent individuals) both recognize RBD distal to RBM and revealed cross-protection against SARS-CoV-2 infection (Pinto et al., 2020; Yuan et al., 2020).

COVID-19 will most likely hit humanity in cycles and gradually disappear, as it has happened for other viral pandemics. Following the 1918 flu pandemic, genetic changes and population immunity ultimately led to the extinction of the 1918 H1N1 lineage decades later (Taubenberger and Kash, 2010). Knowledge of patient zero is still missing, a crucial factor to inform the dynamic of this pandemic. The origins of SARS-CoV-2 are unclear, the virus might be the result of a natural occurrence, yet human adaptation, whether direct from bat or via an intermediate host, is still a matter of speculation (Andersen et al., 2020; Zhang et al., 2020; Zhang and Holmes, 2020). Conclusive evidence on the origin of this pandemic virus is thus essential knowledge still missing (Thorp, 2020a).

## Dissecting the Spike to Seek and Elicit bnAbs

**FIGURE 2:**
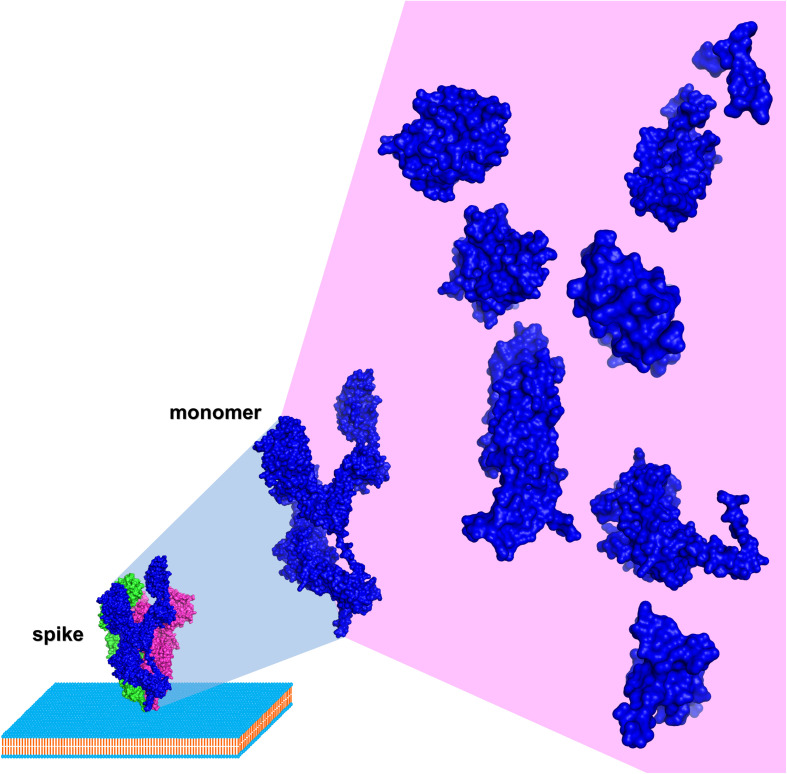
Schematic of HCoV spike dissection (based on SARS-CoV-2, PDB entry 6VYB using PyMOL). Surface representation of a spike monomer (blue) projected from a trimer (blue, red, and green) on a biological membrane, and dissected into autonomously folding subdomains.

HCoV spike sequence analysis and identification of conserved domains distal and within the RBD is the essential rationale of this perspective (Walls et al., 2020; Figure 2). Domain design (by multiple sequence alignment analysis and 3D structure inspection), production and assessment of autonomous folding allow a multiple purposes usage. Simultaneous production of SARS-CoV-2, SARS-CoV, and MERS-CoV variants of the identified domains is instrumental to test broad recognition of nAbs from individuals that survived the infection. Differential testing for bnAbs discovery may initiate from the complete SARS-CoV-2 trimeric spike ectodomain, warranting recognition of authentic epitopes present on the surface of the virus, followed by identification of the binding region using different SARS-CoV-2 spike subdomains and finally testing broad recognition using the homologous domain from different HCoVs. Once identified, these bnAbs could be produced in large quantities, pending the isolation of the memory B cell clones from the individuals producing the bnAbs (Lanzavecchia et al., 2007), providing a first line of passive immunization to be used in an initial phase of a future coronavirus outbreak. Consequently, the molecular localization within isolated spike domains targeted by bnAbs could be the key to an informed strategy toward a broadly neutralizing vaccine, i.e., such domains could be used in the attempt to elicit bnAbs. Notably, spike domains recognized by bnAbs could also be used to screen lead compounds targeting conserved regions as antivirals, e.g., fusion inhibitors targeting the HR1 region in S2 (Xia et al., 2019, 2020).

## Discussion

This perspective highlights the need to look beyond the present urge to defeat COVID-19. Identification, characterization and large scale production of HCoV bnAbs and their conceptual link to a universal vaccine development are essential for an efficacious passive immunization of infected people (bnAbs) and a successful preventative measure to avert other pandemics (universal vaccine). COVID-19 is a reminder that immediate efforts in time of emergency need to be followed by long lasting efforts aimed to prepare for the future (Gudi and Tiwari, 2020; Jacobsen, 2020; Raoult et al., 2020). There is no certainty that we may see an effective SARS-CoV-2 vaccine reaching the public health stage or a drug capable to control the infection. HIV-1 is a dramatic example of a pandemic virus whose molecular organization allowed its escape from a vaccine so far. Influenza virus is a different example since strain-specific vaccines are at reach, yet the risk for the unpredictable emergence of pandemic strains constantly threats human health. In HIV-1, influenza and Ebola virus infection a universal vaccine is mandatory and the last two decades do not provide any reason to omit HCoV from this approach. The exploitation of HCoV spike conserved regions to identify and elicit protective bnAbs is therefore the mainstream to the attainment of an adequate level of preparedness.

## Author Contributions

LV conceptualized the manuscript. LV and MS drafted and finalized the manuscript. All authors approved the submitted version.

## Conflict of Interest

The authors declare that the research was conducted in the absence of any commercial or financial relationships that could be construed as a potential conflict of interest.
